# Reconstruction of chronic insertional tibialis anterior tendon rupture using a semitendinosus autograft with modified side-locking loop suture and multi-anchor fixation: a case report

**DOI:** 10.1186/s13256-026-06082-z

**Published:** 2026-05-13

**Authors:** Junichi Sasaki, Youichi Yasui, Oto Yamamoto, Jun Sasahara, Hirotaka Kawano, Wataru Miyamoto

**Affiliations:** 1 Department of Orthopaedic Surgery, Teikyo University School of Medicine, 2-11-1 Kaga, Itabashi-Ku, Tokyo, 173-8605 Japan; 2 Teikyo University Institute of Sports Science and Medicine, Tokyo, Japan

**Keywords:** Tibialis anterior tendon rupture, Chronic tendon rupture, Tendon reconstruction, Semitendinosus autograft, Side-locking loop suture, Early rehabilitation

## Abstract

**Background:**

Chronic insertional rupture of the tibialis anterior tendon is uncommon and presents substantial reconstructive challenges, particularly when associated with large tendon defects and distal degeneration. Primary repair is often not feasible in such cases, and conventional reconstructive techniques frequently require prolonged postoperative immobilization, delaying functional recovery.

**Case presentation:**

We report a case of chronic insertional tibialis anterior tendon rupture in a 64-year-old Japanese male recreational weightlifter, treated with reconstruction using an ipsilateral semitendinosus autograft. A modified side-locking loop suture technique was employed for proximal fixation, and anatomical distal fixation was achieved using multiple all-suture anchors. This construct was designed to provide sufficient initial biomechanical stability to permit early postoperative rehabilitation. Postoperatively, the ankle was immobilized for 2 weeks, followed by initiation of active range of motion exercises and progressive weight-bearing. At the 2-year follow-up, the patient demonstrated complete pain relief, normal gait, and excellent functional recovery. Isokinetic testing revealed dorsiflexion strength exceeding 95% of the contralateral side, and magnetic resonance imaging confirmed continuity and favorable remodeling of the reconstructed tendon.

**Conclusions:**

This case demonstrates that reconstruction of chronic insertional tibialis anterior tendon rupture using a semitendinosus autograft combined with a modified side-locking loop suture and multi-anchor fixation can provide sufficient stability to allow early functional rehabilitation. This approach may represent a viable treatment option for active patients with large tendon defects.

## Introduction

Atraumatic rupture of the tibialis anterior (TA) tendon is an uncommon clinical entity that presents substantial reconstructive challenges, particularly in chronic cases with delayed diagnosis. When distal degeneration or large segmental defects are present, primary end-to-end repair is often not feasible [1–4].

Although several salvage options—including tendon transfer, lengthening, and interposition grafting—have been reported, these approaches generally require prolonged postoperative immobilization to protect the repair [3, 5–7]. Such immobilization is clinically suboptimal, as it increases the risk of muscle atrophy and delays functional recovery [8].

To address the clinical need for a reconstruction strategy that provides secure fixation while permitting early functional rehabilitation, we report a case of chronic insertional TA tendon rupture treated with a semitendinosus autograft combined with a modified side-locking loop suture (SLLS) and anatomical suture anchor fixation.

## Case presentation

A 64-year-old Japanese male recreational weightlifter presented with a 5-month history of right anterior ankle pain and progressive gait disturbance. Initially diagnosed with TA tenosynovitis at another clinic, he received two corticosteroid injections into the tendon sheath over two months. Although his pain transiently improved, symptoms recurred three months after the last injection, accompanied by an inability to actively dorsiflex the ankle.

**Fig. 1:**
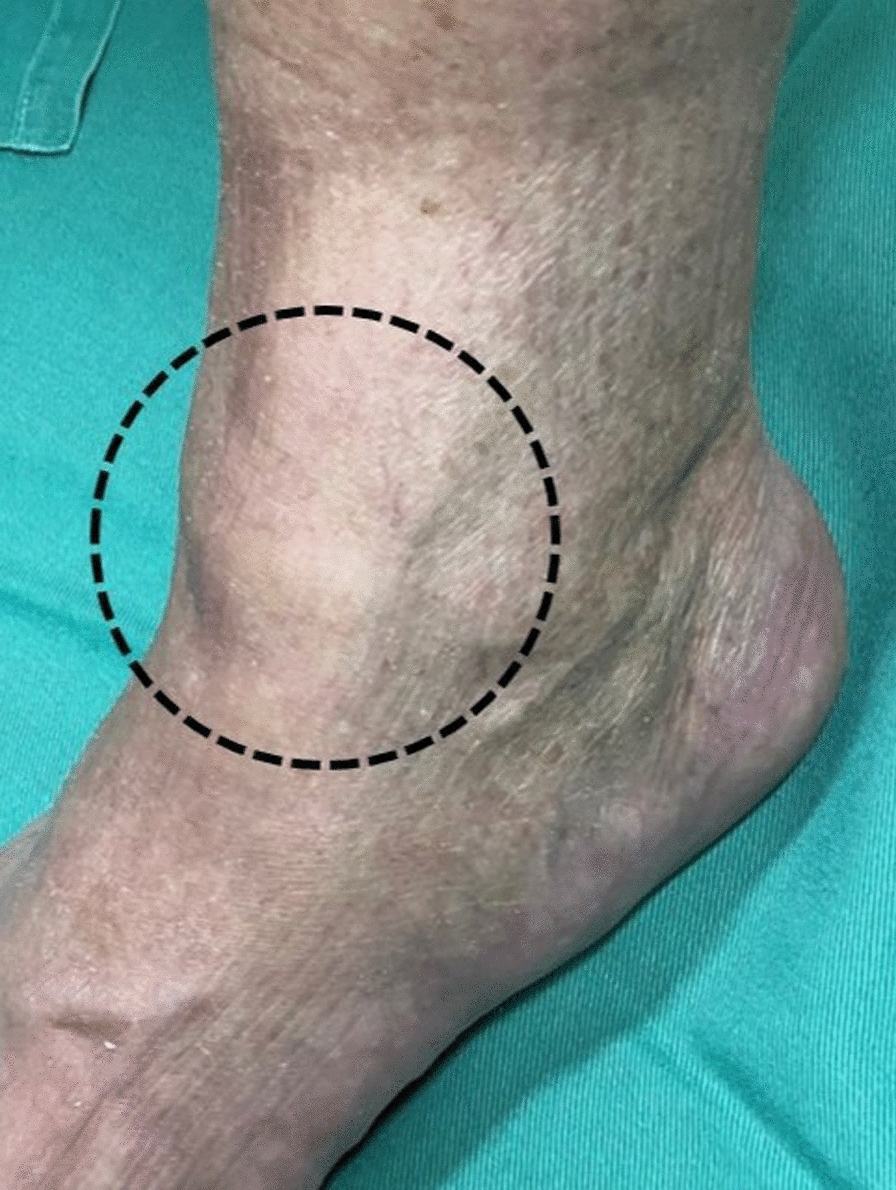
Preoperative clinical photograph of the right ankle. Loss of the normal tibialis anterior tendon contour and a palpable mass at the anteromedial aspect are observed (black-dotted circle)

**Fig. 2:**
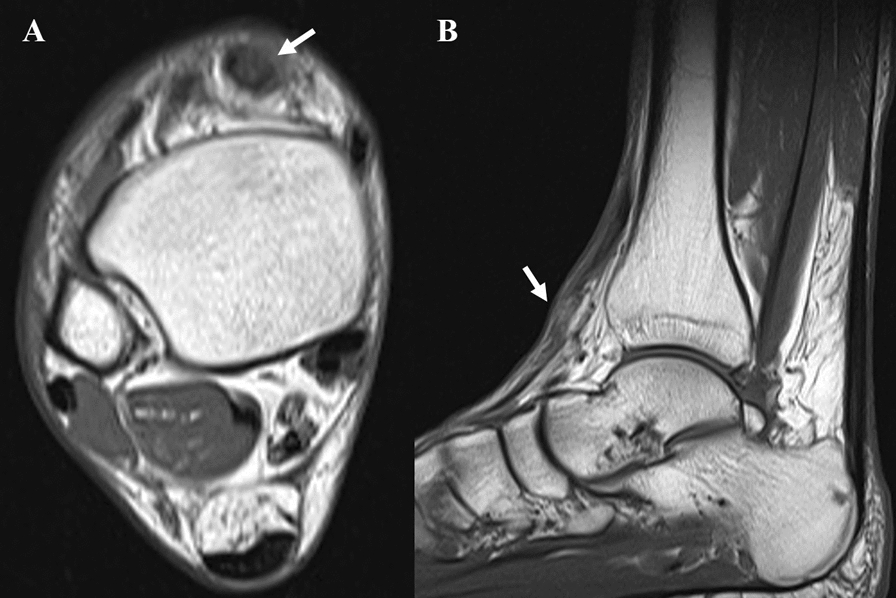
Preoperative magnetic resonance imaging (MRI). **A** Axial and **B** sagittal views showing bulbous expansion of the tibialis anterior tendon at the level of the talocrural joint (white arrows), consistent with degenerative rupture

Physical examination revealed a high-stepping gait and a palpable mass at the anteromedial aspect of the ankle (Fig. 1). Active ankle dorsiflexion was absent (Manual Muscle Test: 0/5), though passive range of motion remained full. There were no sensory deficits. Plain radiographs were unremarkable. Magnetic resonance imaging (MRI) demonstrated a large defect of the distal TA tendon with a bulbous, degenerative proximal stump retracted to the level of the talocrural joint (Fig. 2). Based on these findings, a diagnosis of chronic insertional rupture of the TA tendon was made. Given the significant tendon defect and distal degeneration, primary repair was deemed impossible, and reconstruction using an ipsilateral semitendinosus autograft was selected to permit early mobilization.

### Surgical technique

**Fig. 3:**
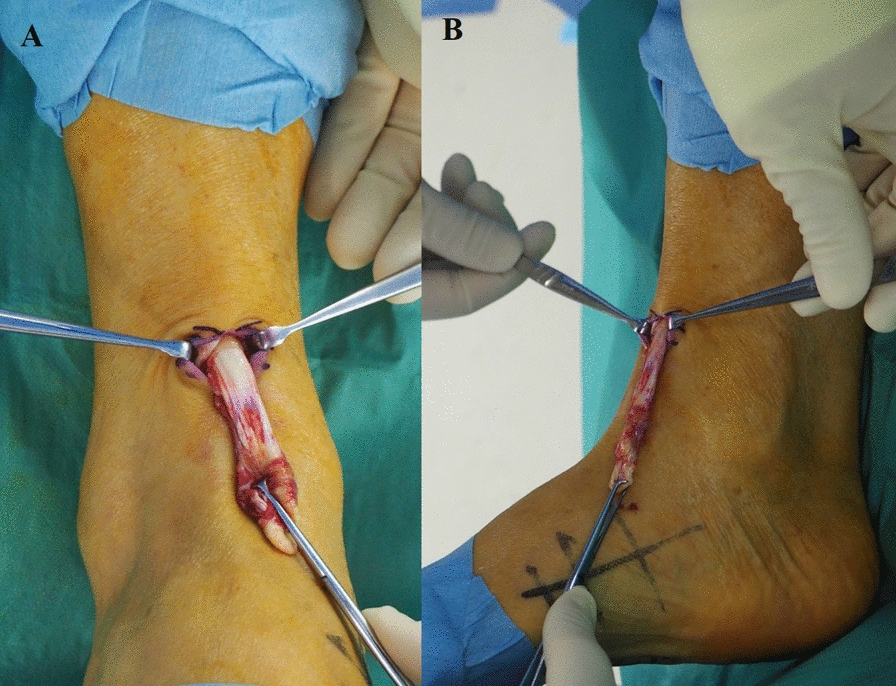
Intraoperative photographs. **A** The proximal tendon stump delivered through the skin incision. **B** The tendon defect after debridement of degenerative tissue. Note that the proximal stump could not be approximated to the insertion site with the ankle in a neutral position

The patient was positioned supine with a thigh tourniquet. A longitudinal incision was made over the distal TA tendon course, preserving the superior extensor retinaculum to prevent bowstringing. The proximal tendon stump was identified, debrided of degenerative tissue, and found to be retracted such that it could not reach the insertion site even with the ankle in neutral (Fig. 3).

**Fig. 4:**
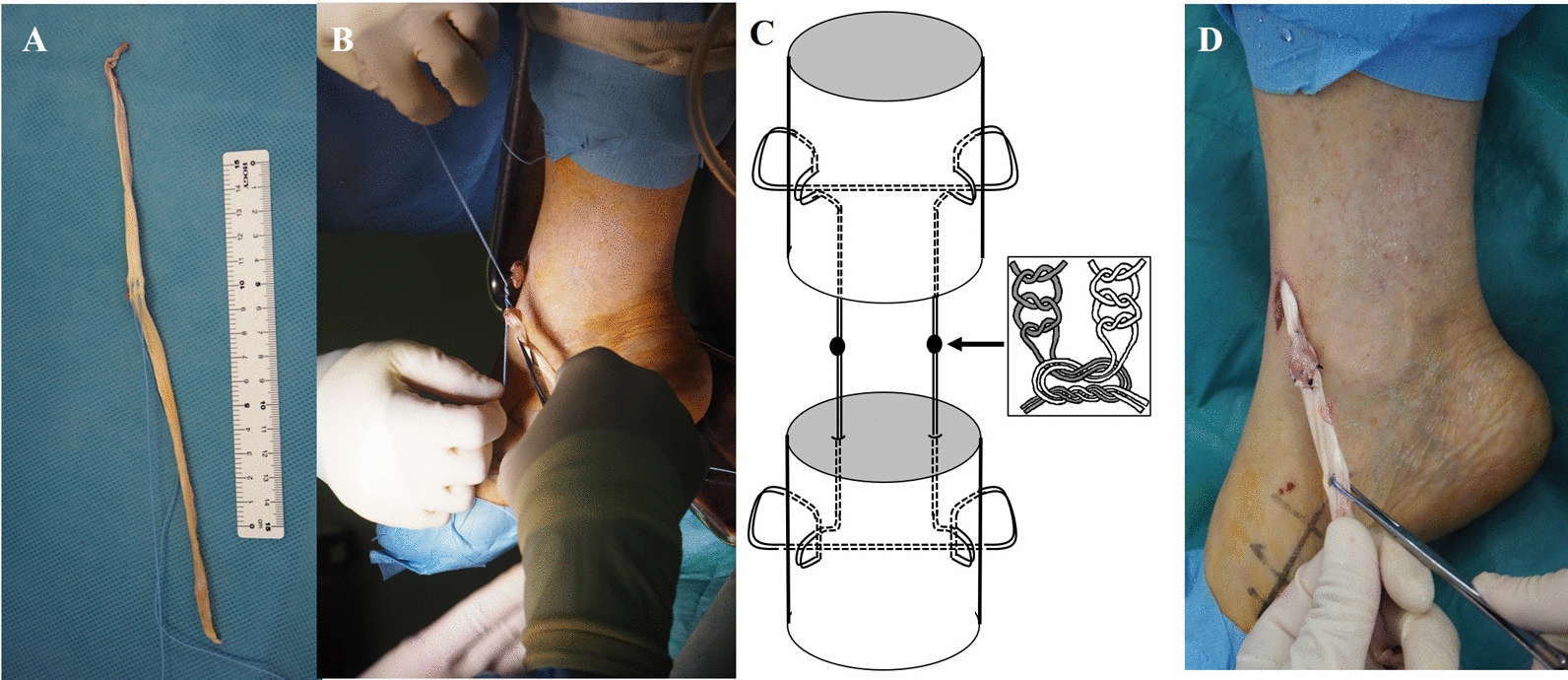
Proximal reconstruction using the modified side-locking loop suture (SLLS) technique. **A** Suturing the blind end of the doubled semitendinosus autograft. **B** Knot tying between the autograft and the native tendon stump. **C** Schematic illustration of the modified SLLS technique. **D** The completed proximal repair reinforced with additional interrupted sutures

**Fig. 5:**
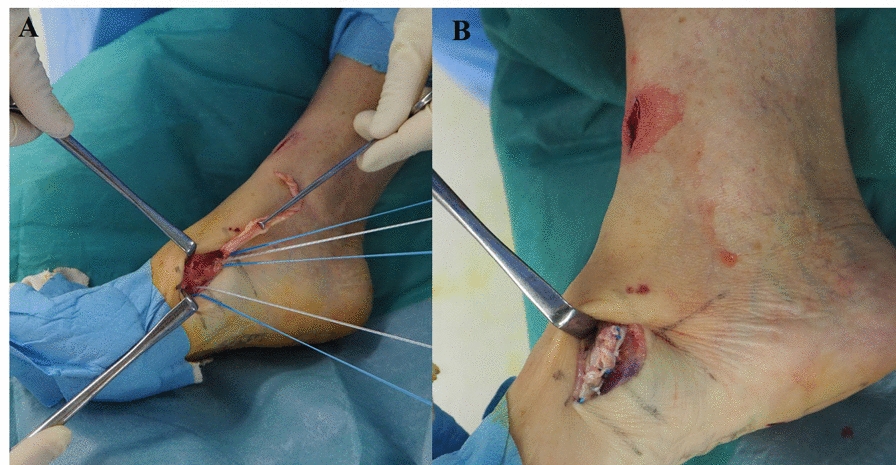
Distal reconstruction. **A** Insertion of suture anchors into the medial cuneiform and the base of the first metatarsal. **B** Completed anatomical reconstruction of the insertion site

An ipsilateral semitendinosus autograft was harvested and doubled. The proximal fixation to the native TA stump was performed using a modified SLLS technique reinforced with No. 2 braided polyethylene/polyester sutures (FiberWire; Arthrex, Naples, FL) (Fig. 4). For distal fixation, the footprint on the medial cuneiform and the base of the first metatarsal was exposed through a second incision. To ensure rigid anatomical reconstruction capable of withstanding early rehabilitation, five 1.8-mm all-suture anchors (Y-Knot; ConMed Linvatec, Largo, FL) were utilized: three in the medial cuneiform and two in the base of the first metatarsal. The graft limbs were tensioned and secured with the ankle held in neutral dorsiflexion (Fig. 5).

### Postoperative course

**Fig. 6:**
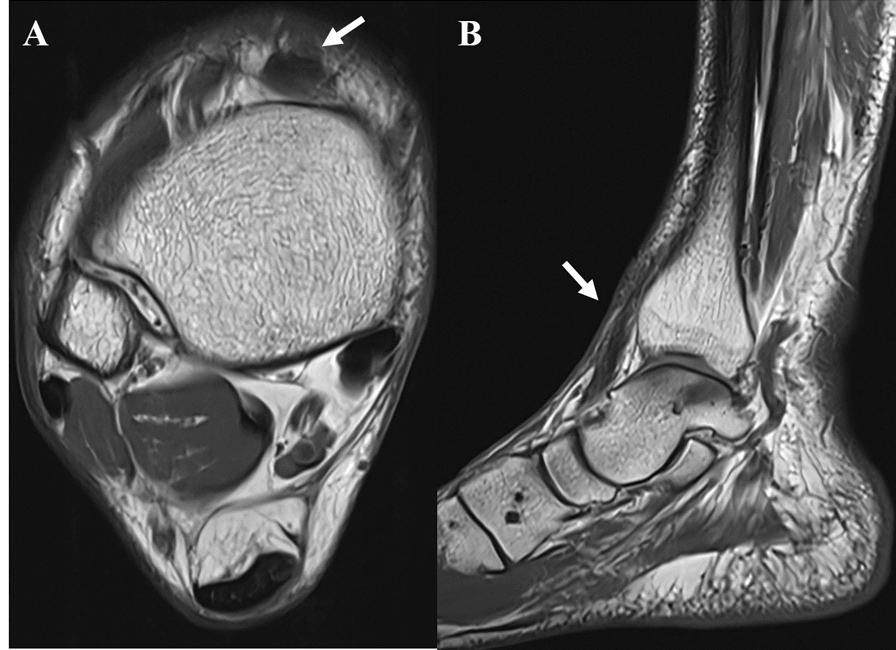
Postoperative magnetic resonance imaging at 1 year. **A** Axial and **B** sagittal views demonstrating the continuity of the reconstructed tendon (white arrows). The graft shows low signal intensity and sufficient bulk, indicating favorable remodeling

The ankle was immobilized in a posterior splint for 2 weeks to allow for soft tissue healing. Active range of motion exercises and partial weight-bearing were initiated immediately after splint removal (2 weeks postoperatively). Full weight-bearing was achieved at 4 weeks. The patient returned to weight training at 3 months. At the 2-year follow-up, the patient was pain-free with no gait abnormalities. Isokinetic testing (Biodex) revealed peak dorsiflexion torque was 96.5% (at 60°/sec) and 97.6% (at 90°/sec) of the contralateral side. The American Orthopaedic Foot & Ankle Society (AOFAS) Ankle–Hindfoot Score [9] was 100, and the Lower Extremity Functional Score [10] was 80. MRI at 1 year confirmed continuity and favorable remodeling of the graft (Fig. 6).

## Discussion

The primary challenge in treating chronic TA tendon ruptures with large segmental defects is bridging the gap while ensuring sufficient biomechanical stability to prevent elongation or re-rupture. While tendon transfer and lengthening are well-established salvage options, they typically necessitate 6 weeks of immobilization, risking muscle atrophy and joint stiffness [10]. In the present case, we utilized an ipsilateral semitendinosus autograft, which offers notable advantages including sufficient length for bridging large defects and a diameter comparable to the native TA tendon [11].

The key feature of our technique is the rigid fixation construct that permitted early rehabilitation. For the proximal repair, we employed the modified SLLS technique [12, 13]. Biomechanical studies have demonstrated that the SLLS provides superior tensile strength compared to traditional suture methods. Komatsu *et al*. reported an ultimate strength of 454 N using No. 2 braided sutures [14], and Yotsumoto *et al*. successfully applied this technique to acute Achilles tendon ruptures without postoperative immobilization, reporting a tensile strength of approximately 900 N [15]. Although the TA tendon is subject to lower physiological loads than the Achilles tendon, this robust suture configuration provides a significant safety margin for early mobilization.

Furthermore, insertional fixation is often considered the "weak link" in reconstruction. To address this, we employed five all-suture anchors, creating a broad, anatomical footprint. Barber *et al.* reported that a single 1.8-mm Y-Knot anchor has a mean ultimate load to failure of 477 N [16]. The use of multiple anchors distributes the load and maximizes pull-out strength, theoretically allowing the construct to withstand the forces generated during early active dorsiflexion.

It is worth noting that the patient had a history of multiple corticosteroid injections. Previous literature suggests a strong correlation between local steroid administration and atraumatic tendon rupture due to collagen degeneration [6, 17]. This case highlights the need for vigilance regarding tendon integrity in patients with a history of injections, particularly when presenting with vague anterior ankle pain.

In conclusion, reconstruction of chronic insertional TA tendon ruptures using a semitendinosus autograft with modified SLLS and multi-anchor fixation provides a biomechanically stable construct. This stability allows for a significantly shortened immobilization period (2 weeks) and early return to activity, making it a viable treatment strategy for active patients with large tendon defects.

## Data Availability

Not applicable.
